# A Rare Case of Palbociclib-Induced Pancolitis: Clinical Insights and Literature Review

**DOI:** 10.7759/cureus.105494

**Published:** 2026-03-19

**Authors:** Khyati Bidani, Abhishek A Chouthai, Arkady Broder, Sugirdhana Velpari

**Affiliations:** 1 Internal Medicine, Saint Peter’s University Hospital, New Brunswick, USA; 2 Gastroenterology and Hepatology, Saint Peter’s University Hospital, New Brunswick, USA

**Keywords:** diarrhea, endoscopy, gastrointestinal toxicity, immune-mediated colitis, pancolitis

## Abstract

Cyclin-dependent kinase 4/6 (CDK4/6) inhibitors are now standard therapies for hormone receptor-positive (HR+), HER2-negative advanced breast cancer. While generally well tolerated, their toxicity profiles differ, with palbociclib and ribociclib more commonly associated with hematologic toxicity and abemaciclib with gastrointestinal adverse effects such as diarrhea. Severe gastrointestinal complications such as colitis remain rare, particularly with palbociclib. We report a 65-year-old woman with metastatic HR+ breast cancer who presented with a two-week history of watery diarrhea (10-12 episodes/day), abdominal cramping, and a 5-pound weight loss while receiving palbociclib, fulvestrant, and denosumab. Infectious studies were negative, and colonoscopy demonstrated moderate pancolitis. Palbociclib was discontinued and intravenous corticosteroids were initiated for suspected drug-induced colitis, resulting in rapid symptom improvement. Although biopsy later demonstrated cytomegalovirus (CMV) positivity, antiviral therapy was deferred given negative CMV PCR and complete clinical resolution. This case highlights an uncommon presentation of palbociclib-induced pancolitis and underscores the importance of careful clinical evaluation and contextual interpretation of histologic findings to avoid unnecessary treatment.

## Introduction

Cyclin-dependent kinase 4/6 (CDK4/6) inhibitors have become a cornerstone in the treatment of hormone receptor-positive (HR+), HER2-negative advanced breast cancer. These agents, including palbociclib, ribociclib, and abemaciclib, work by halting cancer cell proliferation through selective inhibition of CDK4 and CDK6, thereby preventing progression of the cell cycle. When combined with endocrine therapy, CDK4/6 inhibitors have demonstrated significant improvements in progression-free survival, making them an essential component of metastatic breast cancer management [1,2]. Despite their efficacy, these agents are associated with distinct toxicity profiles that can influence patient tolerability and treatment continuation. While hematologic toxicities, such as neutropenia, are more common with palbociclib and ribociclib, abemaciclib is notable for its gastrointestinal (GI) toxicity, particularly diarrhea, which has been reported in up to 90% of patients in clinical studies, although most cases are mild to moderate. In contrast, severe inflammatory gastrointestinal complications such as colitis are rare, with only limited cases described in the literature [2,3,4].

Drug-induced colitis presents a diagnostic challenge as it can mimic inflammatory bowel disease (IBD) and infectious colitis, requiring careful clinical evaluation and exclusion of alternative etiologies [5,6]. In the context of CDK4/6 inhibitors, the clinical presentation, diagnostic approach, and optimal management of colitis remain incompletely characterized. This case report highlights a rare presentation of palbociclib-induced colitis in a patient with metastatic breast cancer, emphasizing the need for vigilance in identifying and managing rare toxicities. By contributing to the growing body of evidence on CDK4/6 inhibitor-associated adverse effects, this report aims to provide valuable insights into the diagnosis and management of severe GI complications, enhancing clinical decision-making.

## Case presentation

A 65-year-old woman with metastatic estrogen receptor-positive breast cancer was admitted to the hospital in December 2024 with a two-week history of watery, non-bloody diarrhea occurring 10-12 times daily, associated with mucus in the stool, cramping abdominal pain predominantly in the lower abdomen with occasional diffuse discomfort, and a 5-pound unintentional weight loss, without associated fever. Based on the stool frequency, the diarrhea was consistent with grade 3 toxicity according to the Common Terminology Criteria for Adverse Events (CTCAE).

Medical history significant for diagnosis with ER-positive metastatic breast cancer in July 2024, decompressive laminectomy for spinal metastases in July 2024, followed by radiation to T9-T11 in August 2024, and initiation of treatment with palbociclib (Ibrance), fulvestrant (Faslodex), and denosumab (Xgeva) in August 2024. Her last dose of Faslodex and denosumab was one day prior to hospitalization, and last dose of Ibrance was seven days prior to hospitalization. There was no prior history of gastrointestinal symptoms, inflammatory bowel disease, or chronic diarrhea.

Investigations

Initial laboratory workup showed a normal white blood cell count and no evidence of neutropenia. Kidney function was preserved with normal creatinine, and liver enzymes were within normal limits. Electrolyte abnormalities, including mild hyponatremia and hypokalemia, were noted and attributed to significant gastrointestinal fluid losses from persistent diarrhea. These were addressed with aggressive electrolyte repletion.

Stool studies, including a bacterial enteric panel, ova and parasites, fecal leukocytes, and Clostridioides difficile testing, were negative. Fecal occult blood testing, however, was positive. Cytomegalovirus (CMV) PCR testing performed on two separate samples was also negative (Table 1).

**Table 1:** Laboratory Results on Admission BUN: Blood urea nitrogen; AST: Aspartate transaminase; ALT: Alanine transaminase; ALP: Alkaline phosphatase; FOBT: Fecal occult blood test; CMV: Cytomegalovirus.

Category	Parameter	Patient Value	Reference Range	Interpretation
Hematology	WBC (×10³/µL)	5.2	4.0 - 11.0	Normal
	Hemoglobin (g/dL)	14.6	12.0 - 16.0	Normal
	Platelets (×10³/µL)	254	150 - 400	Normal
Electrolytes	Sodium (mmol/L)	133	136 - 144	Low
	Potassium (mmol/L)	3	3.5 - 5.1	Low
	Bicarbonate (mmol/L)	20	21 - 33	Low
	Chloride (mmol/L)	111	99 - 112	Normal
Renal Function	BUN (mg/dL)	3	9 - 28	Low
	Creatinine (mg/dL)	0.35	0.52 - 1.04	Low
Liver Function	ALP (U/L)	119	53 - 141	Normal
	ALT (U/L)	18	0 - 35	Normal
	AST (U/L)	19	14 - 36	Normal
	Total Bilirubin (mg/dL)	0.5	0.1 - 1.2	Normal
Stool / Infectious Workup	FOBT	Positive	Negative	Abnormal
	C. difficile PCR	Negative	Negative	Normal
	CMV PCR	Negative	Negative	Normal

Cross-sectional imaging with a CT scan of the abdomen and pelvis with IV contrast revealed no evidence of bowel obstruction, appendicitis, or abscess. Evaluation of the colon for colitis was limited due to the lack of oral contrast. There were postsurgical changes in the thoracic spine and interval sclerosis of a known T10 lytic lesion, consistent with her metastatic disease.

**Figure 1:**
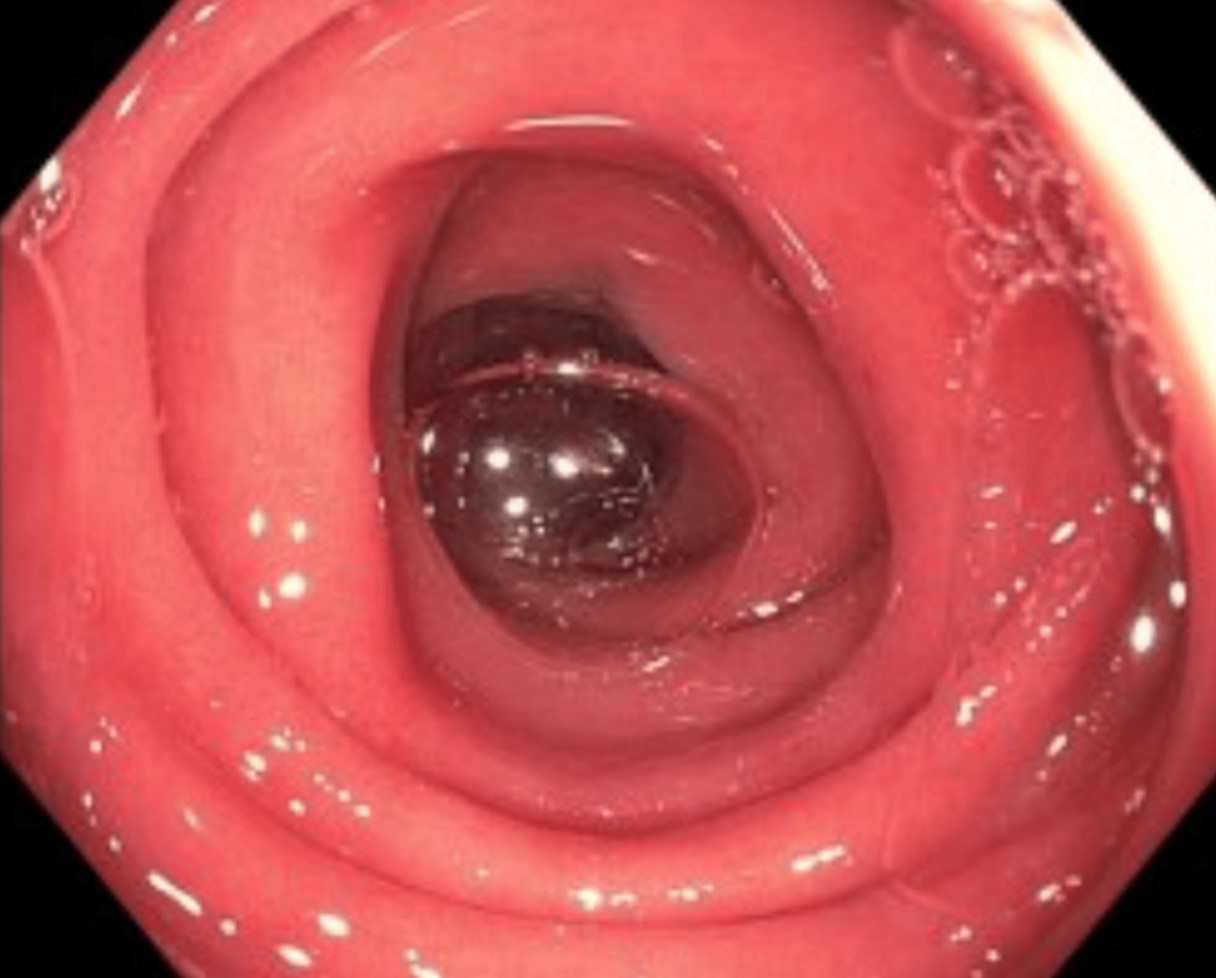
Colonoscopy demonstrating diffuse erythema and loss of vascular pattern consistent with pancolitis.

**Figure 2:**
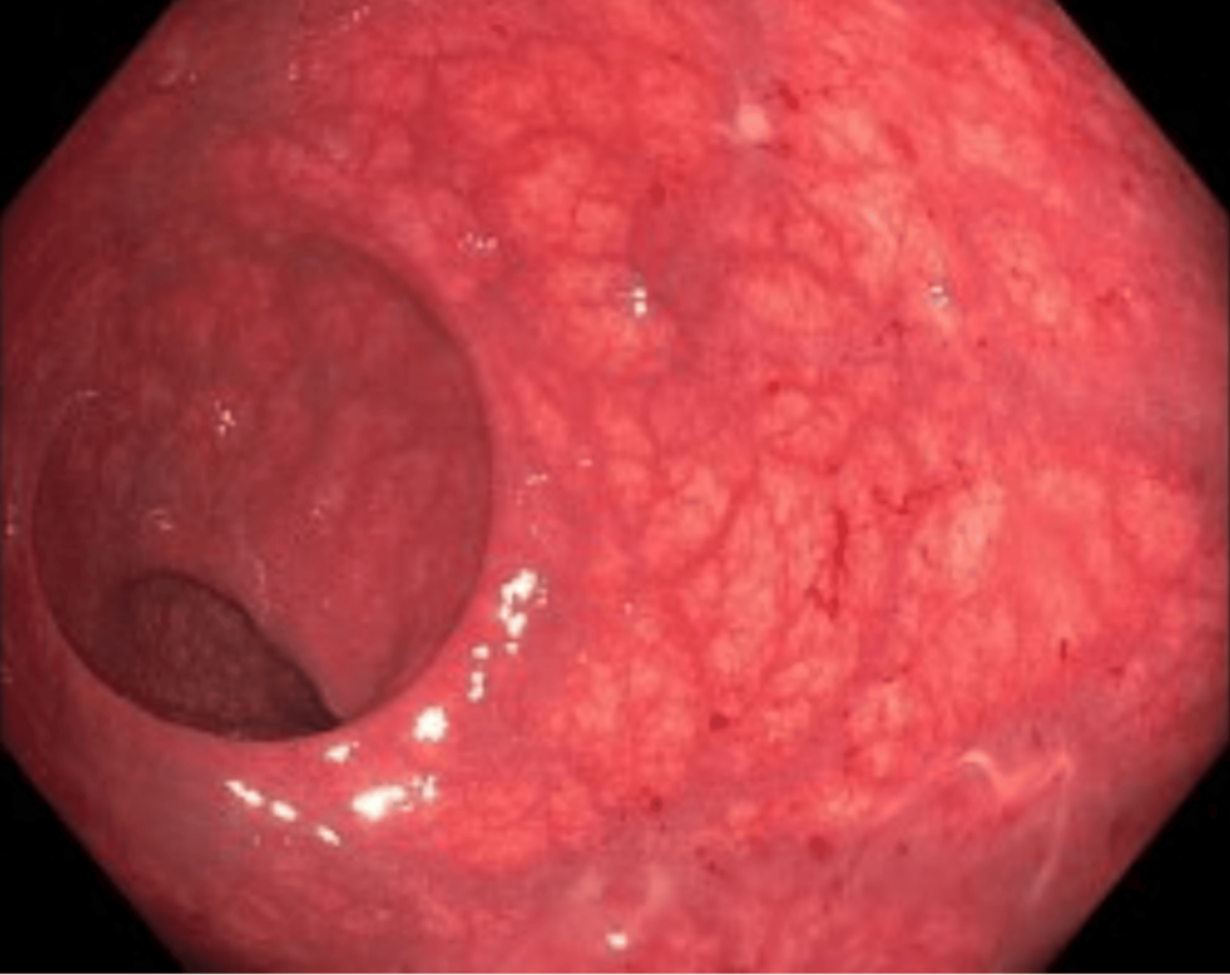
Segmental mucosal hyperemia and edema involving the colon

**Figure 3:**
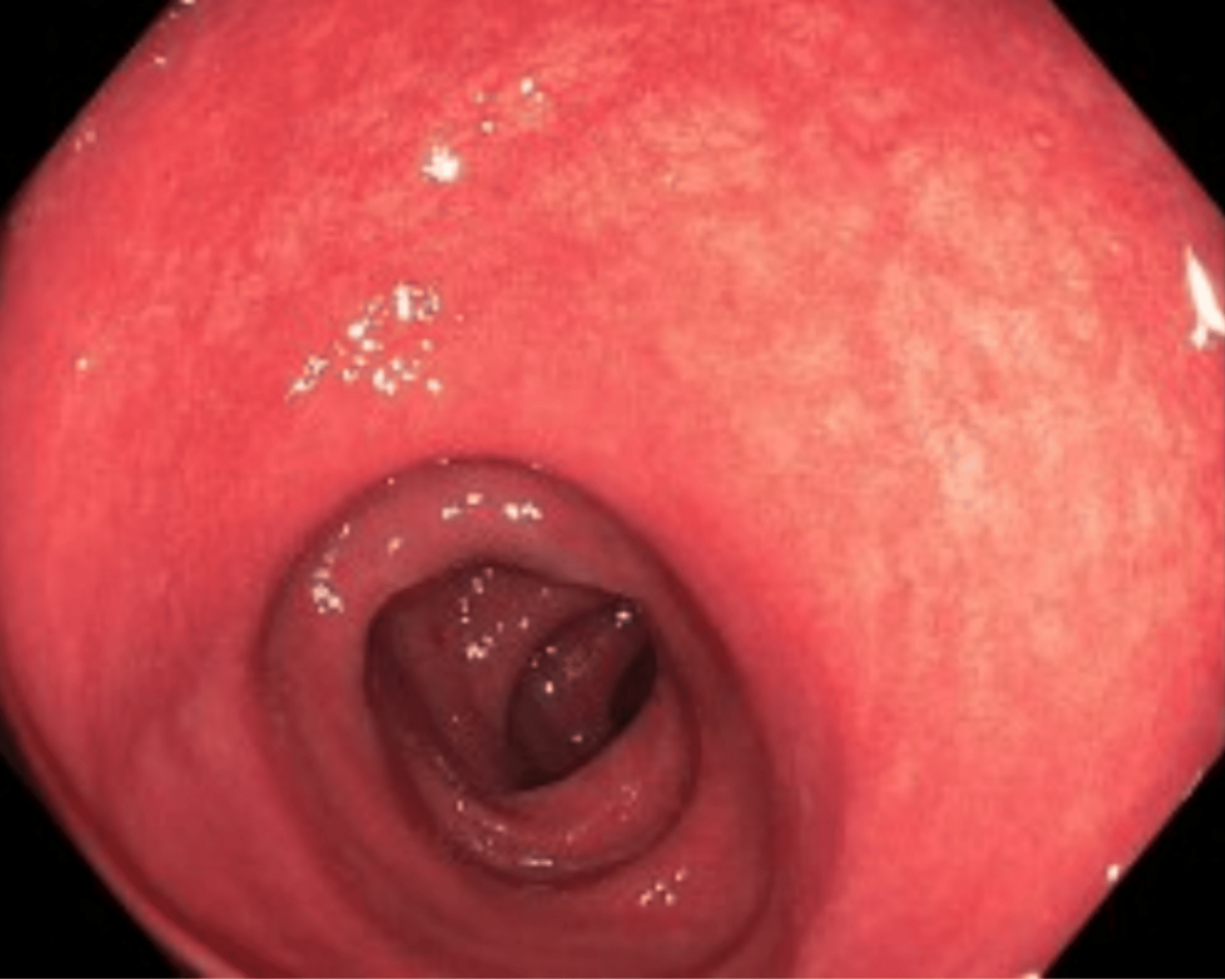
Patchy mucosal inflammation with erythema and friability

Colonoscopy was performed due to the persistence of symptoms despite supportive care. Colonoscopy showed diffuse moderate inflammation characterized by erythema, loss of vascularity, mucus, and erosions in the entire colon consistent with moderate pancolitis (Figures 1-3). Biopsies were taken throughout the colon.

Treatment

Palbociclib was held on admission due to its suspected role in the patient's symptoms. Symptomatic management initially included loperamide 2 mg every 4 hours as needed after infectious causes of diarrhea were ruled out. Electrolyte repletion was performed as needed to address hypokalemia and maintain electrolyte balance.

Given the clinical presentation, endoscopic findings of pancolitis, and lack of infectious etiology, the patient was started on intravenous methylprednisolone at 1 mg/kg, equating to 40 mg twice daily. This regimen led to a rapid improvement in her symptoms, with a reduction in diarrhea frequency from 10-12 episodes daily to two episodes within 48 hours. Following stabilization, she was transitioned to oral prednisone (80 mg daily) with a planned taper of 10 mg every five days over 40 days. Pantoprazole was initiated for gastroprotection during the steroid course.

Before discharge, the case was discussed with her oncologist, who decided to hold palbociclib indefinitely while continuing fulvestrant and denosumab. The patient was discharged in stable condition on a steroid taper, with close follow-up arranged to monitor for symptom recurrence or taper-related issues. Coordination of care was established with her primary care physician, gastroenterology, and oncology teams to ensure comprehensive management.

Pathology results from colonoscopy biopsies, which became available after the patient’s discharge, demonstrated acute and chronic colitis with acute cryptitis, crypt abscess formation, surface erosions, and viral cytopathic inclusions. Immunohistochemical staining for CMV was positive, findings consistent with CMV colitis. However, CMV PCR testing during hospitalization was negative, and the patient experienced complete resolution of symptoms during outpatient follow-up at the gastroenterology clinic. Given the negative PCR results and clinical improvement without antiviral therapy, CMV infection was considered unlikely to be the primary driver of the colitis, and antiviral treatment was deemed unnecessary. An infectious disease specialist was consulted and concurred with this approach, recommending no further treatment unless the clinical condition changed or symptoms recurred.

## Discussion

Cyclin-dependent kinase 4/6 (CDK4/6) inhibitors, including palbociclib, ribociclib, and abemaciclib, have transformed the treatment landscape for hormone receptor-positive (HR+), HER2-negative advanced breast cancer [1]. These agents inhibit cell cycle progression from the G1 to S phase, halting cancer cell division and tumor proliferation [1]. Despite their efficacy, CDK4/6 inhibitors exhibit distinct toxicity profiles that can significantly influence clinical decision-making. Hematologic toxicities, such as neutropenia, are more frequently associated with palbociclib and ribociclib, whereas abemaciclib has higher rates of gastrointestinal (GI) toxicities, particularly diarrhea [2,3]. This case highlights an uncommon but severe GI toxicity, pancolitis, associated with palbociclib, underscoring the importance of recognizing and managing rare adverse events.

This case illustrates a rare but clinically significant adverse effect of palbociclib: severe colitis presenting as pancolitis. While gastrointestinal toxicities are well-recognized with CDK4/6 inhibitors, colitis remains an infrequent and underreported manifestation. The clinical presentation in this case, characterized by diarrhea, abdominal pain, and pancolitis, closely mimicked inflammatory bowel disease (IBD), complicating the diagnostic process. Accurate diagnosis required thorough exclusion of infectious causes, with stool studies ruling out pathogens, including Clostridioides difficile and cytomegalovirus (CMV). The combination of endoscopic findings of pancolitis and histopathological evidence strongly supported the diagnosis of drug-induced colitis, emphasizing the importance of a systematic diagnostic approach.

Diarrhea is a well-recognized side effect of CDK4/6 inhibitors, with varying incidence and severity depending on the agent. Abemaciclib is most commonly associated with gastrointestinal (GI) toxicities, including diarrhea, with real-world data indicating that up to 92% of patients experience diarrhea of any grade, and 17% develop grade 3 diarrhea [4]. In contrast, palbociclib and ribociclib are less frequently linked to diarrhea, though severe GI manifestations like colitis, as seen in this case, remain a possibility [1]. These findings underscore that while GI toxicities are a class effect, the frequency and severity differ across agents.

The impact of diarrhea on treatment continuation is significant. In real-world studies, permanent discontinuation of abemaciclib due to diarrhea was reported in 10% of patients, higher than the 3% observed in clinical trials​​ [4]. This highlights the challenges in managing toxicities in a real-world setting, where patients often have comorbidities or differences in baseline characteristics compared to trial populations. Similarly, while severe colitis is rare, it represents an important consideration for clinicians managing patients on palbociclib, as prompt recognition and management are critical to avoid treatment interruptions and improve outcomes.

Management strategies for CDK4/6 inhibitor-induced diarrhea emphasize early intervention and supportive care. Initiating antidiarrheal agents such as loperamide at the first sign of loose stools and adhering to guideline-based supportive care are essential​​ [2, 4]. For most cases, these measures are sufficient to control symptoms, but individualized management, including dose adjustments or treatment discontinuation, may be necessary in severe cases. In this patient, early identification of colitis, cessation of palbociclib, and corticosteroid therapy led to rapid symptom resolution, highlighting the importance of a tailored approach in managing CDK4/6 inhibitor-induced toxicities.

Two previously published case reports provide additional context for CDK4/6 inhibitor-induced colitis. Malik et al. described ribociclib-induced pancolitis in a 72-year-old woman with metastatic breast cancer, presenting with severe diarrhea and abdominal pain​ [5]. Another report by Gomez et al. detailed ileocolitis in a 30-year-old woman treated with palbociclib, with endoscopic findings resembling Crohn’s disease​ [6]. Both cases required drug discontinuation and corticosteroid therapy, leading to symptom resolution. The current case adds unique insights, including delayed onset of colitis, occurring five months after starting palbociclib, in contrast to the median onset of diarrhea with CDK4/6 inhibitors, such as abemaciclib, typically reported at 6-12 days after treatment initiation [7]. Additionally, this patient demonstrated rapid clinical improvement with corticosteroids despite biopsy findings suggesting CMV colitis, underscoring the importance of clinical correlation when interpreting histopathologic results.

This case report emphasizes the importance of tailoring CDK4/6 inhibitor therapy to individual patient profiles, considering both efficacy and toxicity. As highlighted by comparative studies, the safety profiles of these agents differ due to variations in pharmacokinetics and CDK binding affinities. Palbociclib's intermittent dosing schedule and lower association with GI toxicity make it generally well-tolerated, but rare severe toxicities like pancolitis underscore the need for vigilance. Proactive patient education, close monitoring, and early intervention are essential to optimizing outcomes.

Further research into the mechanisms underlying CDK4/6 inhibitor-induced colitis is warranted to improve early detection and refine management strategies. Additionally, systematic reporting of such rare adverse events can provide valuable insights for developing guidelines and enhancing pharmacovigilance efforts. This case contributes to the growing body of evidence on the rare but significant GI toxicities of CDK4/6 inhibitors, offering important insights for clinicians managing patients on these therapies.

## Conclusions

Palbociclib, though generally well-tolerated, can rarely lead to severe GI toxicity such as pancolitis. Prompt recognition, drug cessation, and systemic corticosteroids led to a full recovery in this case. Clinicians should consider drug-induced colitis in the differential diagnosis of diarrhea in patients receiving CDK4/6 inhibitors. Further pharmacovigilance and reporting of such cases are essential to improve management guidelines and outcomes.
